# GRHAL1, a novel lncRNA, regulates HIV-1 gene expression by modulating Tat- and Sp1-mediated HIV-1 LTR activation

**DOI:** 10.1099/jgv.0.002288

**Published:** 2026-07-02

**Authors:** Mohammad Ishaq, Heather Marshall, Ven Natarajan

**Affiliations:** 1 Laboratory of Molecular Cell Biology, Leidos Biomedical Research Inc, Frederick National Laboratory for Cancer Research, P.O. Box B, Frederick, MD, 21702, USA

**Keywords:** GADD34, gene expression, HIV, HIV-Tat, lncRNA, Sp1

## Abstract

We recently reported that GADD34, an integrated stress response (ISR)-associated protein expressed at low levels in many cell lines, functions as a novel HIV-1 restriction factor. To elucidate the mechanism underlying GADD34-mediated inhibition of HIV replication, we investigated the role of long non-coding RNAs (lncRNAs). Here, we identify and characterize a novel lncRNA, GRHAL1 (GADD34-regulated HIV accessory lncRNA-1), which is highly expressed in GADD34-knockout (GADD34-KO) cells relative to WT cells. GRHAL1 is expressed at low basal levels in Jurkat and MT-2 CD4^+^ T cell lines and in primary human CD4^+^ T cells and is upregulated following HIV-1 infection. T cell activation signals that promote HIV-1 replication also induced GRHAL1 expression; however, this induction was independent of IFN and ISR signalling. Transfection of *in vitro* – synthesized GRHAL1 significantly enhanced HIV-1 LTR-driven gene expression from both integrated and unintegrated promoters. GRHAL1 stimulated both Tat-independent and Tat-dependent LTR activation, with a more pronounced effect observed at suboptimal Tat levels, indicating functional synergy between GRHAL1 and Tat under limiting Tat conditions. Mutational analysis of the LTR demonstrated that GRHAL1-mediated activation requires an intact Tat-responsive TAR element and upstream Sp1-binding sites, linking GRHAL1 activity to Tat and Sp1 in transcriptional regulation. This synergy was further confirmed using a minimal HIV-1 promoter containing Sp1 sites and a TAR sequence. Moreover, GRHAL1 alone was sufficient to activate a heterologous promoter containing Sp1-binding elements. The Sp1-selective inhibitor mithramycin suppressed both Tat-independent LTR activation and the cooperative activation mediated by GRHAL1 and Tat. Electrophoretic mobility shift assays, RNA immunoprecipitation and RNA-pulldown experiments demonstrated that GRHAL1 directly interacts with Sp1 and that Tat enhances this interaction, suggesting that GRHAL1 modulates Sp1 activity through direct binding. In summary, we identify GRHAL1 as a novel HIV-1-induced lncRNA that regulates viral gene expression by interacting with Sp1 and synergizing with Tat to enhance HIV-1 transcription.

## Introduction

In addition to the HIV accessory proteins, HIV-1 is dependent on host cellular factors to replicate and survive [1,3]. Several crucial host factors for HIV-1 biology have been identified in the last few decades. With the recent discovery of thousands of long non-coding RNAs (lncRNAs), there has been a paradigm shift in identifying the role of host factors in the HIV-1 life cycle [4,5]. In fact, several lncRNAs that regulate HIV-1 replication have been identified. For example, lncRNAs HEAL [6] and MALAT1 [7] promote HIV-1 replication, whereas NEAT1 [8], NRON [9] and AK130181 [10] interfere with viral replication.

We recently reported that GADD34, an integrated stress response (ISR)-associated protein that is generally expressed at low levels in many cell lines, is a novel HIV-1 restriction factor and that its function as a restriction factor is independent of its PP1-binding and p-eIF-2α-phosphatase activity [11]. To understand the mechanism of GADD34-mediated inhibition of HIV replication, we sought to identify the role of lncRNAs. Here, we report the identification of a novel lncRNA named GRHAL1 (GADD34-regulated HIV accessory lncRNA-1) that is upregulated in GADD34-knockout (GADD34-KO) cells compared to WT cells. We found that the expression of GRHAL1 is induced after HIV-1 infection and T cell activation but is independent of ISR and IFN signalling. GRHAL1 regulates HIV-1 gene expression by directly binding with the Sp1 protein and synergizing with Tat in the transcriptional activation of the HIV-1 promoter.

## Methods

### Cells and reagents

HEK 293 FT (Thermo Fisher Scientific, Cat. # R70007), HeLa-CD4^+^ [12] and TZM-bl (NIH AIDS Reagent Program, Division of AIDS, NIAID, NIH) cells were maintained in Dulbecco’s modified Eagle medium containing 10% FBS and, for HeLa-CD4^+^ cells, 500 µg ml^−1^ G418. MT-2 cells [13] (NIH AIDS Reagent Program) and Jurkat cells (ATCC, Clone E6-1, Cat. # TIB-152) were maintained in RPMI 1640 medium containing 10% FBS. De-identified PBMCs were procured from healthy donors at the NIH Clinical Center Blood Bank (Clinical Protocol NCT00001846). The cells obtained by lymphapheresis were purified by Ficoll density gradient centrifugation. CD4^+^ T cells were isolated from PBMCs using a Dynabeads™ Untouched Human CD4 T Cells Kit (Thermo Fisher Scientific, Cat. # 11352D) and treated with anti-CD3/CD28 Dynabeads (Thermo Fisher Scientific, Cat. # 11161D) and human recombinant IL-2 (50 U ml^−1^) (MilliporeSigma, Cat. # GF333). The cells were maintained in the presence of IL2 in RPMI 1640 medium containing 10% FBS. IFNβ1 was purchased from R and D Systems (Cat. # 8499-IF) and used at a concentration of 100 ng ml^−1^. Antibodies to GADD34, specific for amino acids 307–572, were obtained from Proteintech (Cat. # 10449–1-AP) and used at 1 : 1,000 dilution. Antibodies to Sp1 used in Western blot were obtained from Sigma Aldrich (Cat. # 07–645) and were used at 1 : 1,000 dilution. Antibodies to Sp1 used in RNA immunoprecipitation (RIP) and specific for amino acids 121–345 near the N-terminus of Sp1 were obtained from Santacruz (Cat. # sc-17824). MG132 (Cat. # M7449 used at 7.5 µM), G418 (Cat. # G418-RO, used at 500 µg ml^−1^), mithramycin A (MA) from *Streptomyces plicatus* (Cat. # M6891, used at 250 nM), phorbol 12-myristate 13-acetate (PMA) (Cat. # P1585 used at 50 ng ml^−1^), ionomycin (ION) (Cat. # AABH9A95673F, used at 1.0 µg ml^−1^) and tunicamycin (TN) (Cat. # T7765, used at 2.5 µg ml^−1^) were from MilliporeSigma. Blasticidin (Cat. # A1113903, used at 10 µg ml^−1^), TURBO DNA-*free™* Kit (Cat. # AM1907), MEGAclear™ Kit (Cat. # AM1908) and Dynabeads™ His-Tag Isolation and Pulldown beads (Cat. # 10104D) were from Thermo Fisher Scientific. Recombinant His-tagged and non-tagged Sp1 protein was from OriGene (Cat. # TP760592) and Active Motif (Cat. # 81181), respectively. His-tagged recombinant HIV-Tat protein (Cat. # 00110 V) was from Virogen, and human testis RNA (Cat. # 636533) was from Takara Bio.

### Gene array analysis for LncRNA expression

RNA from HeLa-CD4^+^ and GADD34-KO HeLa-CD4^+^ cells was analysed for lncRNA expression using a custom Arraystar Human LncRNA microarray. The platform profiles ~39,000 lncRNAs curated from major public databases (e.g. GENCODE, RefSeq, NONCODE, FANTOM5) and large-scale RNA-seq datasets. Each transcript is represented by exon- or splice junction-specific probes, with built-in positive and negative controls for quality assessment. Array images were processed using Agilent Feature Extraction software (v11.0.1.1), and data were quantile-normalized and analysed with GeneSpring GX v12.1. Differentially expressed lncRNAs were identified using fold-change and volcano plot filtering criteria.

### 5′ and 3′ rapid amplification of cDNA ends and cloning of GRHAL1

5ʹ and 3ʹ rapid amplification of cDNA ends (RACE) analysis of RNAs was performed using the SMARTer RACE 5′/3′ Kit (Takara Bio) using total RNA from Jurkat and MT-2 cells. GRHAL1 was cloned in pcDNA3.1 vector (Thermo Fisher Scientific).

### Generation of CRISPR-mediated GADD34-knockout cells

CRISPR-mediated GADD34-KO HeLa-CD4^+^ cells were generated by AcceGen Biotechnology. The deleted region was a 3,201 bp fragment spanning exons 2 and 3 of the GADD34 gene. The gRNA sequences used were GGACTATGACTTTGTCGCCAAGG and ACTGACAGCATTCTACTTACAGG.

### RNA isolation and real-time PCR

Total RNA was isolated using the Qiagen RNeasy kit according to the manufacturer’s protocol and treated with DNase to remove residual genomic DNA. RNA was reverse-transcribed using SuperScript IV reverse transcriptase (Thermo Fisher Scientific) with 2.5 µM random hexamers. cDNA was analysed by real-time PCR (RT-PCR) using gene-specific primers (Table 1) on a 7500 Fast Real-Time PCR System with Fast SYBR Green Master Mix (Applied Biosystems) or TaqMan probes (Integrated DNA Technologies). No-reverse transcriptase controls were included to confirm the absence of DNA contamination. RNase P and GAPDH served as reference genes for normalization, and relative expression was calculated using the comparative Ct method.

**Table 1.** List of primers

RT-PCR primers	
AJ011932.1/F	GCGGAAACCGCATGTGTAAC
AJ011932.1/R	TCTACTGCCTCCACTATCCAGT
AC090204.1/F	CCCAGCTAGAGTGTGGAAGC
AC090204.1/R	TCTGGTGAATTGGACCCGAAC
XLOC_014244 /F	CCTTCAACTCCACGGGACAA
XLOC_014244 /R	ATTCACCAGGCATCCTCAGC
AC138904.1/F	GGGGATTTCCCCAGCATGTC
AC138904.1/R	GGACCAACAAGCCAGGAATG
GRHAL1/F	CATTCACACTCCGCAAACAG
GRHAL1/R	GTGAACCCATCTCCATCTCC
GRHAL1-probe	/56-FAM/AG GTG CCC AGC AAA CAC GC/36-TAMSp/
TAT-F	AGACAGCGACGAAGAGCTCATCAG
TAT-R	CCACCTTCTTCTTCTATTCCTTCGG
RNASEP-F	AGATTTGGACCTGCGAGCG
RNASEP-R	GAGCGGCTGTCTCCACAAGT
GAPDH-F	TGACAACTTTGGTATCGTGG
GAPDH-R	ATGATGTTCTGGAGAGCCC
DDIT3/F	TAAAGATGAGCGGGTGGCAG
DDIT3/R	GCTTTCAGGTGTGGTGATGT
LOC101928796/exon 1 F	TCAGCGCCGTCCCTTCCCGGGAAGCCTCCT
LOC101928796/exon 2 R	TGGGCGACCTCGGCGATCCAGGAATCGGTC
**5′ RACE primers**	
GRHAL1-5′ GSP1/R	CCTGTACGGCCAGTGGAAGTGGCACAGGGACAG
GRHAL1-5′-GSP2/R	AAGGGGTGCACGCGGCCCTCGGCTCCTGTCCCG
**3′ RACE primers**	
GRHAL1-3′-GSP1/F	TATTCCTGCCAGATGTTCAGAGGCGGAGGCCAG
GRHAL1-3′-GSP2/F	TGTGTTTTCCTATCTAGGAGTCAGTCTGTCGCA
***In vitro* transcription primers**	
GRHAL1-sense/F	AAAATAATACGACTCACTATAGGGAGAGAGAGGGCGGGAACGC
GRHAL1-sense/R	GTGGAACAAAGACTTTTTTTATTTCTTTCTAC
GRHAL1-antisense/F	GAGAGGGCGGGAACGC
GRHAL1-antisense/R	AAAATAATACGACTCACTATAGGGAGAGTGGAACAAAGACTTTTTTTATTTCTTTCTAC

### Western blot

Protein extracts were electrophoresed in a 4–12% NuPAGE Bis-Tris Gel using NuPAGE MES SDS running buffer (Thermo Fisher Scientific), then transferred to a PVDF membrane using an XCell Blot Module (Thermo Fisher Scientific). After treatment with primary antibodies, protein was detected using fluorophore-labelled secondary antibodies and the Odyssey Infrared Imaging System (LI-COR Biotechnology). Protein-specific bands were normalized to total proteins. Total proteins were stained using SimplyBlue SafeStain (Thermo Fisher Scientific) or Revert 700 Total Protein Stain (LI-COR Biotechnology), per the manufacturers’ instructions, and the stained gels or membranes were scanned using the Odyssey Infrared Imaging System.

In some experiments, protein expression was quantified using the Jess automated capillary-based Simple Western system (ProteinSimple, Bio-Techne). Cells were lysed in M-PER Extraction Reagent (Thermo Fisher Scientific) supplemented with protease and phosphatase inhibitors. Total protein concentration was determined using the BCA Protein Assay Kit (Thermo Fisher Scientific). Lysates were diluted in 0.1X sample buffer and mixed with fluorescent 5X master mix and 40 mM DTT, both from the manufacturer, followed by denaturation at 95 °C for 5 min. Samples (3 µl) were loaded onto a 12–230 kDa size-based separation cartridge with fluorescence detection (Bio-Techne, #SM-FL004). Proteins were separated by capillary electrophoresis, immobilized to the capillary wall via UV activation and incubated sequentially with primary antibody against Sp1 (1 : 250 dilution, Sigma-Aldrich, #07–645) and fluorescently labelled secondary antibodies (Bio-Techne, #042–206). Fluorescent signals were detected and electropherograms were generated using the instrument’s fluorescence module. Peak integration and quantification were performed using Compass for Simple Western software (v7.0.0). Protein abundance was calculated from baseline-corrected peak area values and normalized to total protein signal measured in the integrated Protein Normalization Module (Protein Simple #DM-PN02) to account for capillary-to-capillary loading variation.

### Viral infections

HIV-1 pNL4-3 (an infectious molecular clone of HIV-1) plasmid was used for generating HIV-1 in HEK 293 FT cells, as previously described [11]. pNL4-3 plasmid [14] was obtained from the NIH AIDS Reagent Program. HIV-1 infection was performed in HeLa-CD4^+^, Jurkat, MT-2 and human CD4^+^ cells by spinoculation with 20–40 ng of CA-p24-antigen-equivalent HIV-1. HIV-1 in the supernatants was quantitated at the indicated times using the Alliance HIV-1 CA-p24 Antigen ELISA kit from PerkinElmer.

### *In vitro* transcription and transfections

GRHAL1 RNA and GRHAL1-antisense RNA were synthesized from PCR products by T7 polymerase-mediated transcription using a MEGAscript™ T7 Transcription Kit Plus from Thermo Fisher Scientific. PCR products were generated from cloned GRHAL1 plasmid. For generating sense DNA templates by PCR, the forward primer contained the T7 promoter sequence immediately upstream of the transcription initiation site, and for generating antisense DNA templates, T7 promoter sequences were included at the 5ʹ end of the reverse primers (Table 1). RNA was synthesized using 1 µg of purified PCR product, treated with TURBO DNAse and purified using a MEGAclear™ kit, per the manufacturer’s instructions.

pBlue3′LTR-luc-A (containing luciferase driven by HIV-1 3′LTR) and pNL4-3.Luc.R-E-plasmids were from the NIH AIDS Reagent Program as previously described [11]. pN3-SP1FL plasmid was from Addgene (Cat. #24543). The Sp1-nonexpressing pN3-SP1-STOP plasmid was generated by introducing premature stop codons at amino acid positions 4 and 8 within the Sp1 ORF of the pN3-SP1FL plasmid. pcDNA-Sp1-FLAG was from Addgene (Cat. #232649). Control *FLAG* plasmid was an in-house plasmid expressing LZTFL1 protein [15]

Cells were transfected with plasmids alone or co-transfected with plasmids and GRHAL1 RNA by using FuGENE-HD (Promega) at a DNA/RNA: FuGENE-HD ratio of 1 : 3. The plasmid–FuGENE-HD complexes were incubated at room temperature for 30 min before being added to the cells. The cells were incubated for 36–65 h depending on the cell type, and luciferase activity was quantitated using the Luciferase Assay System (Promega, Cat. # E1500). The luciferase activity was normalized to total protein concentration in the cell lysates using the BCA Protein Assay Kit (Thermo Fisher Scientific).

### Lentiviruses and transductions

Vesicular stomatitis virus (VSV)-pseudotyped lentiviruses were obtained from Gentarget. VSV-pseudotyped pNL4-3.Luc.R-E- lentivirus was prepared using plasmid pNL4-3.Luc.R-E-. GADD34-expressing lentiviral particles and blasticidin selection were used to generate GADD34-KI single-cell clones from GADD34-KO cells. To test the effect of GRHAL1 overexpression on single-round HIV-1 replication, Jurkat cells were transfected with GRHAL1 RNA for 23 h, then transduced with VSV-G-pseudotyped pNL4-3.Luc.R-E- viral particles (1 p.f.u. cell^−1^) for 3 days and luciferase activity was quantitated using the Luciferase Assay System and normalized to total protein concentration in the cell lysates using the BCA Protein Assay Kit.

### Cell proliferation and apoptosis

HIV-1 virus- or heat-inactivated HIV-1 virus-infected Jurkat cells (0.2 million) were seeded in 96-well plates in 100 µl medium. Cell viability was assessed by using 3-(4,5-dimethylthiazol-2-yl)−2,5-diphenyltetrazolium bromide (MTT)-based CellTiter 96 Cell Proliferation Assay Kit (Promega Corporation), as per the manufacturer’s instructions. For apoptosis induction, Jurkat cells were treated with 100, 250 and 500 ng ml^−1^ anti-Fas antibody for 16 h, and cell viability was assessed by MTT assay. Anti-Fas antibody-induced apoptosis was also confirmed by quantitating FasL mRNA, a known marker of Fas-induced apoptosis, by RNA isolation followed by RT-PCR.

### RNA electrophoretic mobility shift assay

Cy5-labelled GRHAL1 RNA probe was generated from 1 µg of GRHAL1 DNA PCR product via a T7-polymerase-mediated *in vitro* transcription assay by using a HighYield T7 Cy5 RNA labelling kit (UTP-based) (Cat. # RNT-101-CY5, Jena Bioscience). RNA was treated with TURBO DNAse and purified using a MEGAclear™ kit, per the manufacturer’s instructions.

For electrophoretic mobility shift assay (EMSA), 25 ng of the Cy5-labelled GRHAL1 probe was incubated with recombinant Sp1 protein using binding buffer (40 mM Tris, pH 8.0; 30 mM KCl; 1 mM MgCl_2_; 1 mM DTT; 0.01% NP-40; 100 ng ml^−1^ yeast tRNA; and 200 µg ml^−1^ heparin) at 4 °C for 30 min. For the competition assay, a 10–20-fold excess of consensus double-stranded (ds) Sp1 DNA oligo or a mutant ds Sp1 DNA oligo or a 25-fold excess of unlabeled GRHAL1 RNA was incubated with recombinant Sp1 protein in the binding buffer at 4 °C for 15 min, after which 25 ng Cy5-labelled probe was added and the samples were incubated at 4 °C for another 15 min. For super-shift assays, 2 µg of anti-Sp1 antibodies (Millipore) was added for an additional 15 min at 4 °C after incubating the recombinant Sp1 protein in binding buffer with 25 ng of Cy5-labelled GRHAL1 probe for 30 min at 4 °C. The shifted bands were resolved on 1% agarose gels, then scanned at the 700 nm channel using a LI-COR Odyssey fluorescent scanner.

### RNA immunoprecipitation

293 FT cells (1×10⁷) were co-transfected with 1 µg pcDNA-GRHAL1 and 1 µg pcDNA-Sp1-FLAG or a control FLAG plasmid. After 48 h, cells were harvested and washed twice with cold PBS supplemented with EDTA-free protease inhibitors. Nuclear extracts were prepared using the Nuclear Extraction Kit (Active Motif, Cat. # 40010) and resuspended in lysis buffer from the kit (50 µl per 8×10⁶ cells) supplemented with EDTA-free protease inhibitors (Halt™ Protease Inhibitor Cocktail, EDTA-Free, Thermofisher, Cat. # 78425). Nuclear extracts were sheared by chilled water bath sonication (QSonica Q800R3) using cycles of 10 s ON and 30 s OFF for a total of 2 min (three rounds). Protein concentration was determined by BCA assay (Pierce). For each immunoprecipitation, 75 µg of nuclear extract was treated with 2 µl (4 U) TURBO DNase at 37 °C for 30 min, followed by pre-clearing with Pierce™ Protein A/G Magnetic Beads (Thermo Fisher, Cat. # 88802) for 15 min at 4 °C with rotation. Anti-FLAG M2 magnetic beads (Millipore, #M8823) were equilibrated in RIP buffer (50 mM Tris/HCl, 150 mM NaCl, 0.05% NP-40, 1 mM MgCl₂) supplemented with RNase inhibitors. Pre-cleared extracts were adjusted to 500 µl in RIP buffer and incubated with 25 µl of equilibrated beads for 2.5 h at 4 °C with rotation. Beads were washed six times with 500 µl RIP buffer. For protein analysis, 100 µl of the final bead suspension was eluted with 30 µl 3×FLAG peptide (Millipore, #F4799; 150 ng µl^−1^) for 15 min at room temperature with rotation. Western was performed using 4 µl of the eluted samples with the Jess automated capillary-based Simple Western system, using 1 : 500 dilution of anti-FLAG antibody (Origene, Cat. # TA50011-100) and fluorescently labelled secondary antibodies (Bio-Techne, Cat. # 042–205). For RNA isolation, the remaining 400 µl bead suspension was incubated in 126 µl RIP buffer, 15 µl 10% SDS and 9 µl Proteinase K (Thermo Fisher, #AM2546) at 50 °C for 30 min with mixing. The mixture was then adjusted to 300 µl with RLT buffer (Qiagen RNeasy Kit, #74104) supplemented with 100 ng HeLa RNA as a recovery control. Input samples (75 µg) were processed in parallel in RIP buffer and RLT buffer. RNA was purified using the RNeasy kit (Qiagen), treated with TURBO DNase and reverse-transcribed using SuperScript IV (Thermo Fisher). Quantitative PCR was performed using gene-specific primers for GRHAL1. RIP enrichment was calculated as the fraction of immunoprecipitated RNA relative to input using the formula 2^(Ct input – Ct IP).

### RNA-pulldown assay

A pulldown assay using Dynabeads™ His-Tag Isolation and Pulldown Kit (Thermo Fisher Scientific) was conducted to confirm the interaction between GRHAL1 and Sp1. For this, 2.5 µg Jurkat nuclear extract (JNE) (Santacruz Cat. # sc-2132) was spiked with 3.5–7.0 pmol of recombinant His-tagged Sp1 and 100 ng of GRHAL1 RNA in a total volume of 500 µl RNA-EMSA buffer (for the buffer, see RNA-EMSA under Methods, above). In experiments where the goal was to study the interaction of the HIV-1 Tat protein with GRHAL1 or to study the effect of the HIV-1 Tat protein on the GRHAL1–Sp1 interaction, 1 µg His-tagged Tat was added to the reaction. The mixtures were incubated at 4 °C for 30 min and transferred to tubes that contained 50 µl of Dynabeads™ His-Tag beads previously separated from the solution using a magnetic rack. The mixture was incubated at 4 °C for an additional 30 min, then the beads were separated using a magnetic rack and washed three times with 500 µl EMSA buffer. GRHAL1 bound to the beads was eluted in RNA-lysis buffer by using an RNeasy kit (Qiagen). For normalization, the eluants were spiked with equal amounts of Rous sarcoma virus (RSV) (particles before RNA isolation). RNA was isolated using the RNeasy kit. Purified RNAs were treated with DNase and subjected to RT-PCR using GRHAL1- and RSV-specific primers.

### Statistical analysis

Data are presented as mean±sd from representative experiments with two to five replicates. Unless otherwise indicated, experiments were independently repeated three times. Statistical analyses were performed using GraphPad Prism (GraphPad Software). Comparisons between two groups were conducted using unpaired two-tailed Student’s *t*-test, while multiple group comparisons were analysed by one-way ANOVA with appropriate post-hoc tests. A *P*-value<0.05 was considered statistically significant (* *P*≤0.05, ** *P*<0.01).

## Results

### Identification and characterization of GRHAL1

To investigate the mechanism of inhibition of HIV-1 replication by GADD34, we investigated the role of lncRNAs, regulatory RNAs that are known to influence every aspect of host-virus biology, and their roles are often context specific and mechanistically informative. The specific role of *previously uncharacterized lncRNAs* directly regulated by GADD34 is still a largely unknown area of research in viral biology. To study the role of GADD34-regulated lncRNAs in HIV-1 gene expression, we generated a GADD34-KO HeLa-CD4^+^ cell line and then examined the expression of lncRNAs from WT and knockout (KO) cells by using a custom gene array.

**Fig. 1.**
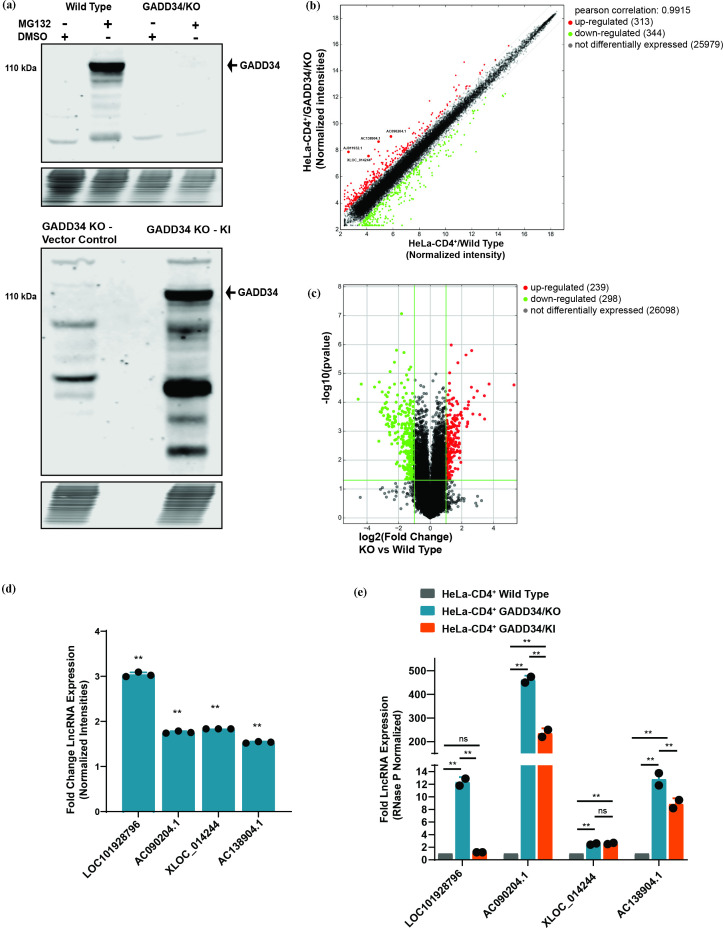
GADD34 regulates lncRNA expression. (**a**) Western blot analysis of GADD34 protein in HeLa-CD4^+^ WT and GADD34-KO cells (upper panel), and in GADD34-KO cells transduced with vector control or GADD34 (KI) (lower panel). MG132 (a proteasome inhibitor) was used to stabilize GADD34 to detectable levels. Induction was observed only in WT cells, confirming the absence of GADD34 expression in KO cells. (**b and c**) RNA from HeLa-CD4^+^ and GADD34-KO HeLa-CD4^+^ cells was analysed in triplicate for lncRNA expression using a custom Arraystar microarray. The data are presented as a scatter plot (**b**) and a volcano plot (**c**), illustrating overall variation and the magnitude of differential lncRNA expression, respectively. Differences in the number of upregulated, downregulated and unchanged transcripts reflect the more stringent statistical threshold applied in the volcano plot. (**d**) Gene array data showing four highly upregulated lncRNAs (LOC101928796, AC090204.1, XLOC_014244 and AC138904.1), presented as fold change compared to HeLa-CD4^+^ WT, set to 1. (**e**) Restoration of lncRNA expression following GADD34 KI in KO cells was assessed by RT-PCR quantification, normalized to RNase P.

Successful KO of GADD34 was confirmed by treatment with MG132, a proteasome inhibitor that enhances GADD34 stability [16]. MG132 treatment enabled detection of GADD34 protein in WT cells, which otherwise express low basal levels not readily detectable by Western blot, whereas no GADD34 expression was observed in KO cells (Fig. 1a, upper panel). A raw scatter plot showed 313 lncRNAs were upregulated, 344 lncRNAs were downregulated and 25,979 lncRNAs were not differentially expressed in GADD34-KO cells compared to WT cells (Fig. 1b). A volcano plot (Fig. 1c), based on log2 fold change identified 239 lncRNAs were upregulated, 298 ncRNAs were downregulated (Fig. S1, available in the online Supplementary Material) and 26,098 lncRNAs were not differentially expressed. We subsequently validated the expression of several GADD34-regulated lncRNAs. Among the validated lncRNAs, four that were highly upregulated in GADD34-KO cells in the gene array screen – AJ011932.1 (hereafter referred to by its NCBI designation LOC101928796), AC090204.1, XLOC_014244 and AC138904.1 (Fig. 1d) – were further evaluated for their expression following GADD34 reconstitution in GADD34-KO cells. GADD34 knock-in (KI) (Fig. 1a, lower panel) restored the LOC101928796 lncRNA (NCBI designation: LOC101928796), which was highly upregulated in GADD34-KO HeLa CD4^+^ cells, to the levels seen in WT HeLa CD4^+^ cells (Fig. 1e), strongly indicating that GADD34 mediated LOC101928796 expression and was therefore selected for further studies.

**Fig. 2.**
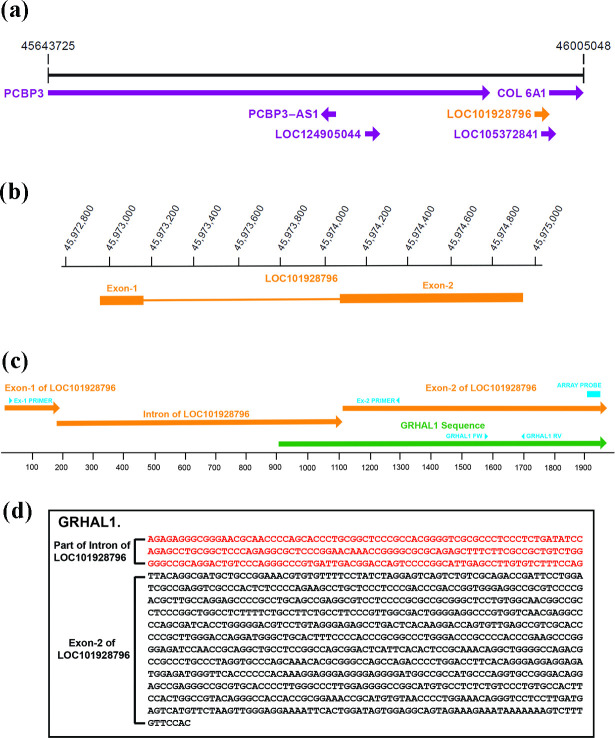
Characterization of GRHAL1. (**a and b**) AJ011932.1 (LOC101928796) RNA was identified as an uncharacterized intergenic, 1,067-nucleotide-long lncRNA with two exons and an intron located between the *PCBP3* and *COL6A1* genes in chromosome 21. (**c**) Location of the newly characterized lncRNA GRHAL1. GRHAL1 lacked exon 1 of LOC101928796 lncRNA but instead contained an additional 213 bases of intron from LOC101928796 gene. The probe used for the lncRNA array screen was specific to exon 2 of lncRNA LOC101928796 and is shown. The location of exon 1, exon 2 and GRHAL1-specific primers is shown. (d) DNA sequence of GRHAL1. Sequence highlighted in red (1–213 bases) is the extended exon 2 of LOC101928796 lncRNA and the 214–1072 is the exon 2 of lncRNA LOC101928796.

LOC101928796 was identified as an uncharacterized 1,067-nucleotide-long RNA with two exons with enriched expression in human testis. The NCBI database (https://www.bi.nlm.nih.gov/gene/101928796#gene-expression) shows that there is a biased expression in testis (RPKM 1.6), indicating higher expression in testis than most other tissues. Although PCR primers specific for exon 1 and exon 2 readily detected lncRNA LOC101928796 (Fig. 2a and b) in total RNA from human testis, these primers failed to detect this lncRNA in HeLa-CD4^+^, Jurkat, MT-2 and peripheral blood CD4^+^ T cells (Fi). The probe used for the LncRNA array screen was specific to LOC101928796 exon 2 (Fig. 2c). 5′ and 3′ RACE analyses of Jurkat and MT-2 cellular RNA identified an isoform of lncRNA LOC101928796 that lacks exon 1 and instead contains an extended exon 2, with an additional 213 nucleotides extending into the upstream intron of the LOC101928796 gene (Fig. 2c). We designated this isoform, the major one expressed in Jurkat and MT-2 cell lines, as GRHAL1 (Fig. 2d). GRHAL1 is an intergenic lncRNA located between the PCBP3 and COL6A1 genes (Fig. 2a). Later studies indicated that GRHAL1, in addition to the lncRNA LOC101928796, is also expressed in human testis (Table 2S).

### GRHAL1 is induced by HIV-1 infection

**Fig. 3.**
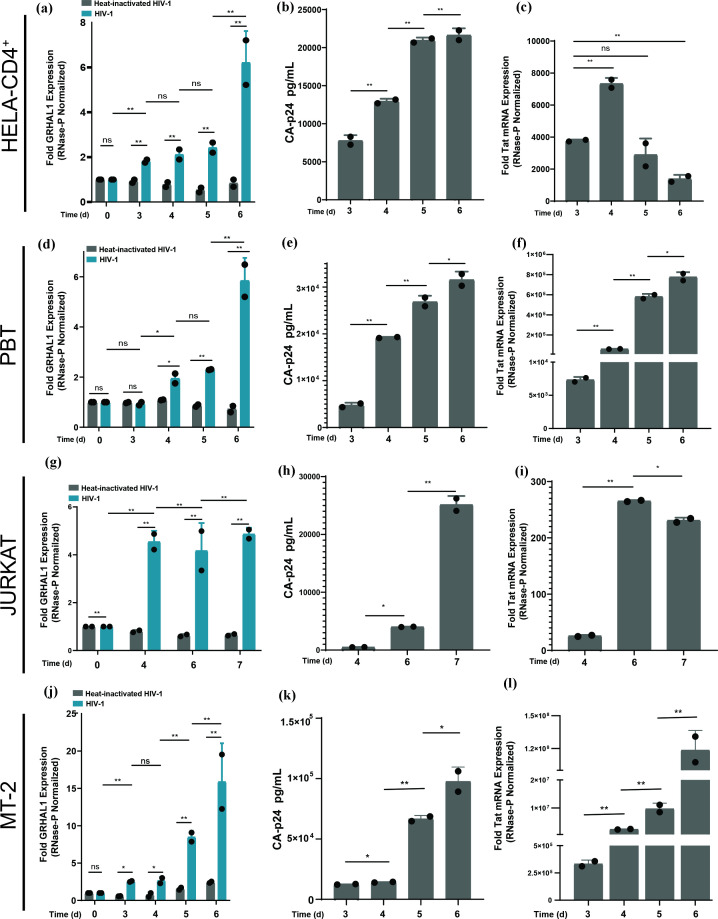
GRHAL1 is induced by HIV-1 infection. (a–l) Kinetics of GRHAL1, CA-p24 and HIV-1 Tat mRNA expression following HIV-1 infection in HeLa-CD4^+^ (a–c), human peripheral blood CD4^+^ T (PBT) (d–f), Jurkat (g–i) and MT-2 cells (j–l). Cells were infected by spinoculation with 20–40 ng of CA-p24 antigen equivalent HIV-1. Heat-inactivated virus was used as control. HIV-1 in the culture medium was quantitated at the indicated times using the Alliance HIV-1 CA-p24 Antigen ELISA kit from PerkinElmer. GRHAL1 and HIV-1 Tat RNAs in cell pellets were quantitated by RT-PCR and normalized to RNase P.

To explore the role of GRHAL1 in HIV-1 infection, we studied GRHAL1 expression kinetics after HIV-1 infection in C (Fig. 3a–c), human CD4^+^ T (Fig. 3d–f), Jurkat (Fig. 3g–i) and MT-2 cells (Fig. 3j–l). In the physiological state, all these cells express low basal levels of GRHAL1 but showed a progressive increase in GRHAL1 after HIV-1 infection in HeLa-CD4^+^, human CD4^+^ T and MT-2 cells, coinciding with an increase in the accumulation of CA-p24 protein. GRHAL1 expression was induced by 3 days post-infection, and the levels peaked ~6–7 days post-infection. In Jurkat cells, however, the kinetics of GRHAL1 expression post-HIV-1 infection was different, with an increase on day 4 that remained stable till day 7. This may be due to relatively rapid HIV-1 signal response and sensing in Jurkat cells, where GRHAL1 levels saturate earlier, whereas CA-p24 and Tat response is proportional to the viral burden. In HeLa-CD4^+^ cells, we noticed that Tat mRNA levels decreased at later time points with increasing CA-p24 and GRHAL1 levels. Although HeLa-CD4^+^ cells are HIV-1 permissive, but unlike physiological CD4^+^ T cells, regulatory kinetics of Tat mRNA may differ in HeLa-CD4^+^ cells. In these cells, declining *Tat* mRNA with increasing CA-p24 protein may reflect a shift in *HIV transcriptional regulation* and *RNA processing*.

To exclude the possibility that GRHAL1 induction was not due to cell death caused by HIV infection, we infected Jurkat cells with HIV-1 up to 7 days and monitored cell death using MTT assay. Jurkat cells were found to be resistant to HIV-1-induced cell death (Fig. S3), indicating that HIV-1-induced GRHAL1 upregulation is not due to cell death but due to active HIV-1 sensing.

### GRHAL1 is induced during T cell activation, but its expression is independent of IFN and ISR signalling

**Fig. 4.**
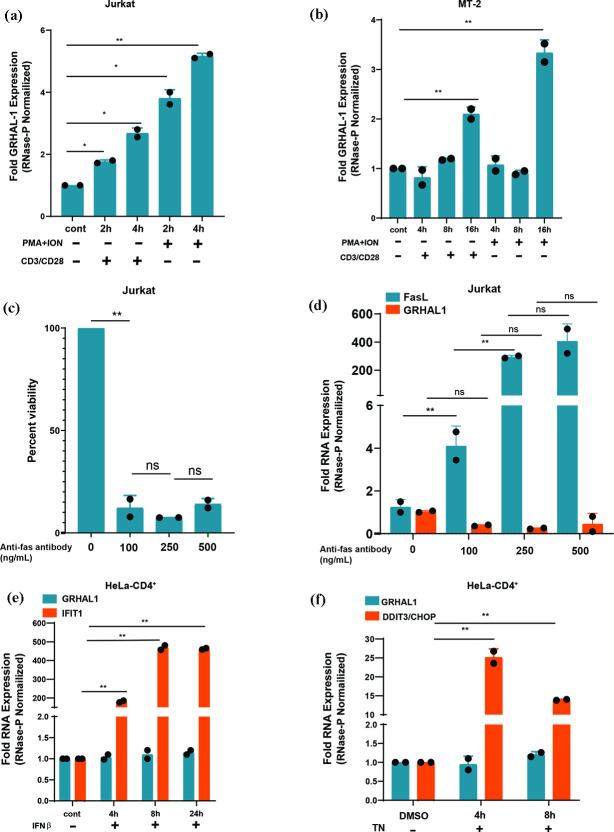
GRHAL1 is induced during T cell activation, but its expression is independent of IFN and ISR signalling. (**a and b**) Jurkat (**a**) and MT-2 (**b**) cells were treated with anti-CD3 and CD28 beads or PMA (50 ng ml^−1^) and ION (1 µg ml^−1^) for the indicated time periods. Cells were harvested for RNA isolation and RT-PCR with GRHAL1-specific primers normalized to RNase P. (**c and d**) Cell proliferation assay and RT-PCR quantitation of GRHAL1 and FasL mRNA using anti-Fas antibody-treated Jurkat cells. Jurkat cells were treated with the indicated concentrations of anti-Fas antibodies for 16 h and subjected to MTT assay (**c**) and RT-PCR for indicated RNAs (**d**). HeLa-CD4^+^ cells were treated with IFNβ1 (**e**) or TN (**f**) for the indicated time periods. Cellular RNA was isolated and RT-PCR was performed to estimate GRHAL1 and IFIT1 (**e**) or GRHAL1 and DDIT3/CHOP RNAs (**f**). IFIT1 and DDIT3/CHOP, which are induced by IFN-1 and TN treatment, respectively, were used as controls to confirm the efficacy of the treatments. All the RT-PCR data were normalized to RNase-P.

The engagement of T cells by T cell receptors (TCRs), CD28 and other T-cell-activating agents has been shown to enhance HIV-1 replication predictably through several host signalling mechanisms that influence replication [17,19]. To study the effects of T cell activation on the expression of GRHAL1, we treated Jurkat (Fig. 4a) and MT-2 cells (Fig. 4b) with antibodies to CD3 and CD28 or PMA+ION and followed the expression of GRHAL1. Both treatments resulted in a significant induction of GRHAL1 expression, indicating an overlap between TCR signalling and the signalling mechanisms that define GRHAL1 expression. To exclude the possibility that GRHAL1 induction during T cell activation was not due to activation-induced apoptosis, we treated Jurkat cells with different doses of anti-Fas antibodies, known to induce apoptosis, and monitored cell viability and the GRHAL1 expression. As can be seen in MTT assay (Fig. 4c), treatment with anti-Fas antibodies induced marked cell death in these cells and showed a dose-dependent induction of apoptosis marker FasL (Fig. 4d). However, there was no significant effect of apoptosis on the expression of GRHAL1 (Fig. 4d). Together, these data show that GRHAL1 induction is independent of cell death and apoptosis.

We have shown that GRHAL1 expression is regulated by GADD34 (Fig. 1b–e), an IFN-stimulated gene (ISG) that is also induced by the ISR pathway [20,21]. To study the role of IFN and ISR signalling in the regulation of GRHAL1 expression, we treated HeLa-CD4^+^ cells with IFNβ1 and TN, reagents that are known to induce IFN and ISR-mediated signalling, respectively [22,23]. Neither IFNβ1 (Fig. 4e) nor TN (Fig. 4f) treatment induced GRHAL1 expression, indicating that GRHAL1 expression is independent of IFN and ISR signalling and that GADD34-mediated regulation of GRHAL1 expression may not involve these pathways. IFIT1 and DDIT3/CHOP are known to be induced by IFNβ1 and TN treatment, respectively, and were used as controls to confirm the efficacy of the treatments.

### GRHAL1 induces Tat-independent and Tat-dependent activation of both the unintegrated and integrated HIV-1 LTR promoter and induces HIV-1 replication

**Fig. 5.**
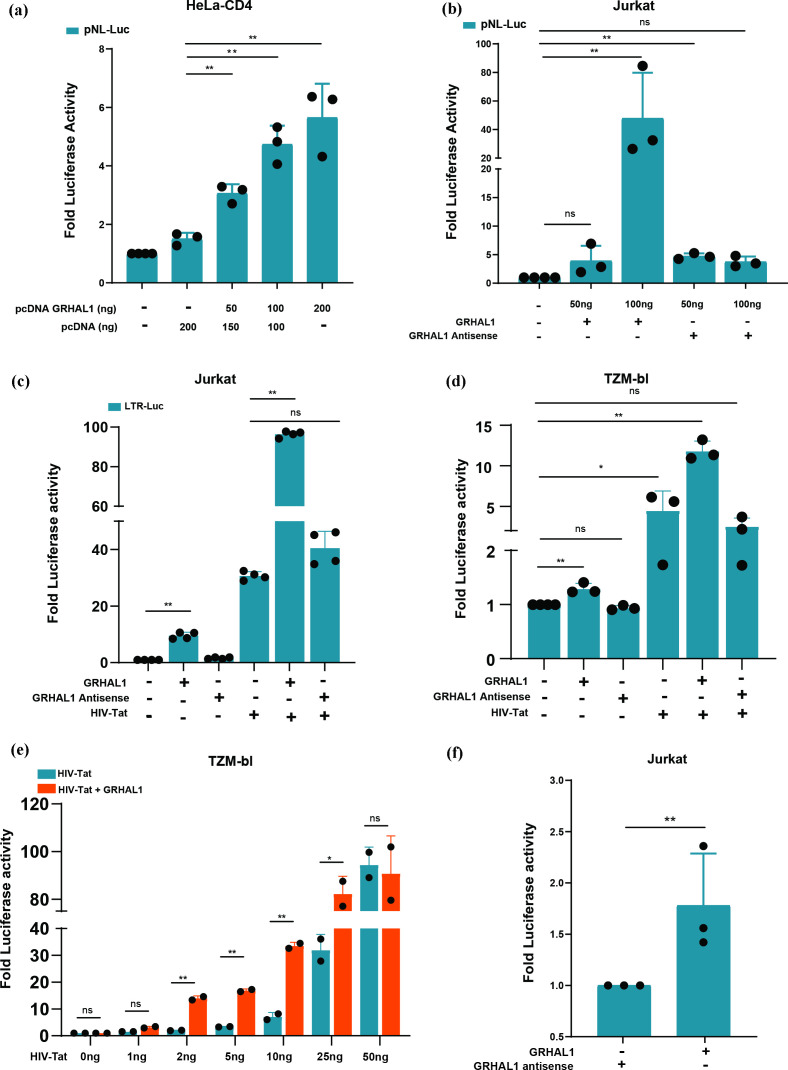
GRHAL1 induces Tat-independent and Tat-dependent activation of both the unintegrated and integrated HIV-1 LTR promoter. (**a**) HeLa-CD4^+^ cells (0.1 million) were co-transfected with 100 ng pNL4-3.Luc.R-E- plasmid in the presence of indicated concentrations of pcDNA3.1 vector or pcDNA3.1–GRHAL1 plasmid. Cells were harvested 36 h later for luciferase assay. (**b**) Jurkat cells (0.25 million) were co-transfected with 0.25 µg of pNL4-3.Luc.R-E- plasmid and indicated concentrations of GRHAL1 RNA or GRHAL1-antisense RNA. Cells were harvested after 65 h for luciferase assay. (**c**) Jurkat cells (0.25 million) were co-transfected with 0.25 µg of pBlue-LTR-Luc plasmid in the presence or absence of HIV-1 Tat plasmid (100 ng) and indicated concentrations of GRHAL1 RNA or GRHAL1-antisense RNAs. Cells were harvested after 65 h for luciferase assay. (**d**) TZM-bl cells (0.1 million) were co-transfected with indicated concentrations of GRHAL1 RNA or GRHAL1-antisense RNAs in the presence or absence of HIV-1 Tat plasmid. Cells were harvested after 36 h for luciferase assay. (**e**) Effect of different concentrations of Tat on the GRHAL1-induced LTR activation. TZM-bl cells (0.1 million) were co-transfected with increasing concentrations of HIV-1 Tat plasmid in the presence of GRHAL1 RNA. Cells were harvested after 36 h for luciferase assay. (**f**) To test the effect of GRHAL1 overexpression on single-round HIV-1 replication, Jurkat cells were transfected with GRHAL1 RNA for 16 h, then transduced with VSV-G-pseudotyped pNL4-3.Luc.R-E- viral particles (1 p.f.u. cell^−1^) for 3 days and luciferase activity was quantitated.

Because our data have shown that HIV-1 infection induces GRHAL1 expression, we sought to study the effect of GRHAL1 on HIV-1 gene expression. Co-transfection of HeLa-CD4^+^ cells with a plasmid expressing GRHAL1 (GRHAL1 cloned in pcDNA3.1, a cytomegalovirus-promoter-containing plasmid vector) and a replication-incompetent pNL4-3.Luc.R-E- plasmid showed an increase in the luciferase activity dependent on pcDNA3.1–GRHAL1 concentration (Fig. 5a). We found that, when compared, co-transfection of Jurkat cells with pNL4-3.Luc.R-E- plasmid in the presence of in-vitro-synthesized GRHAL1 RNA was much more efficient in inducing luciferase activity than co-transfection with pNL4-3.Luc.R-E- and pcDNA3.1/GRHAL1 plasmids (Fig. 5-S1), perhaps due to higher efficiency of RNA delivery and the rapid transient effect of the RNA. Similar results were seen in HeLa-derived cell lines (Fig. 5-S2). There was no toxicity associated with the transfections as there was no effect on the protein levels in the transfected lysates (Fig. 5-S3). Co-transfection of Jurkat cells with GRHAL1 RNA in the presence of pNL4-3.Luc.R-E- plasmid showed a significant concentration-dependent increase in the luciferase activity as compared to the GRHAL1-antisense RNA used as a control (Fig. 5b). The range of induction was dynamically broad leading up to a 50-fold induction at 100 ng of the GRHAL1 sense RNA as compared to antisense RNA. This broad range could reflect some unknown coactivator limitations in various experiments. In some experiments, we also observed minor activity from GRHAL1 antisense RNA suggesting that unlike *sense* RNA, antisense RNA may not contain potential sequence/structural motifs that recruit activating cofactors in trans when delivered to the cell or antisense activity is mediated by a different, low-efficiency mechanism that may involve weak recruitment of an activator resulting in minor activity.

To study whether GRHAL1 RNA induces both Tat-independent and Tat-dependent activation of the HIV-1 LTR promoter, Jurkat cells were co-transfected with pBlue3′LTR-luc-A plasmid, a plasmid in which luciferase activity is driven by HIV-1 3′-LTR, and GRHAL1- RNA or GRHAL1-antisense RNA in the presence and absence of Tat-expressing plasmid. GRHAL1 RNA significantly induced both Tat-independent and Tat-dependent activation of HIV-1 LTR promoter, whereas the GRHAL1-antisense RNA showed no effect on activation (Fig. 5c). Similar data were seen with TZM-bl cells (Fig. 5d), a HeLa-cell-derived luciferase reporter cell line [24] that harbours an integrated copy of the HIV-1 LTR promoter and is engineered to express CD4, CCR5 and CXCR4 [25]. However, the effect of GRHAL1 RNA on Tat-independent activity was lower in TZM-bl cells than in Jurkat cells. To study the effect of Tat on GRHAL1-induced LTR activation, we transfected TZM-bl cells with increasing concentrations of Tat plasmid in the presence of GRHAL1 RNA. GRHAL1 RNA induced LTR activation only at suboptimal levels of Tat, and the effect of GRHAL1 was lost at higher concentrations of Tat (Fig. 5e). In summary, these data demonstrate that GRHAL1 induces Tat-independent and Tat-dependent activation of both the unintegrated and integrated HIV-1 LTR and that GRHAL1–Tat synergy is seen only at suboptimal concentrations of Tat.

To test the effect of GRHAL1 overexpression on single-round HIV-1 replication, we transfected Jurkat cells with GRHAL1 RNA for 16 h, then transduced them with VSV-G-pseudotyped pNL4-3.Luc.R-E- viral particles (1 p.f.u. cell^−1^) for 3 days. GRHAL1 RNA induced nearly 1.8-fold increase in the luciferase activity as compared to GRHAL1 antisense control, indicating that GRHAL1 overexpression induces a moderate but statistically significant increase in the HIV-1 replication (Fig. 5f).

### GRHAL1-mediated activation of HIV LTR is dependent on an intact Tat-binding TAR region and Sp1-binding promoter elements

**Fig. 6.**
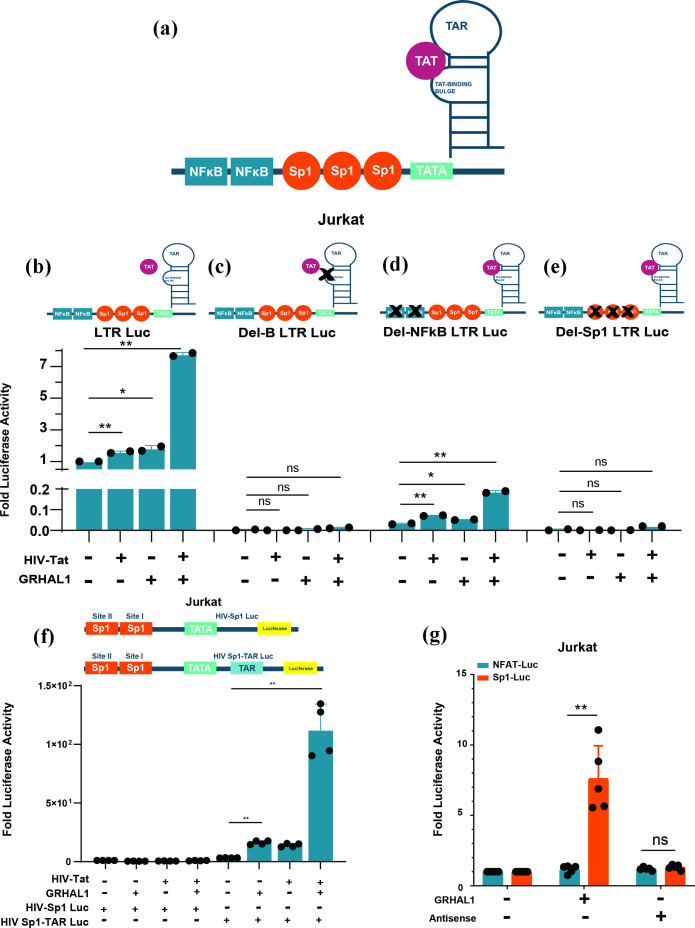
GRHAL1-mediated activation of HIV LTR is dependent on an intact Tat-binding TAR region and Sp1-binding promoter elements. (**a**) HIV-1 LTR promoter core region showing the location of two NF-κB-binding and three Sp1-binding sites and the conserved TAR RNA hairpin structure with the Tat-binding bulge region downstream of the TATA box. (b–e) Jurkat cells (0.25 million) were co-transfected with 250 ng of pBlue3′LTR-luc plasmid (**b**), Tat-binding bulge deletion mutant Del-B-LTR-Luc plasmid in which TTT (+22 to +24) were deleted (**c**), Sp1-deleted LTR-Luc plasmid in which three Sp1 sites were deleted (**d**), or NF-κB-deleted LTR-Luc plasmid in which two NF-κB sites were deleted (**e**), with all the transfections performed in the presence of suboptimal levels of Tat and 100 ng of GRHAL1 RNA. The cells were harvested for luciferase assay 65 h after transfection. (**f**) Jurkat cells (0.25 million) were co-transfected with 500 ng of plasmids (that contained two LTR-specific Sp1 sites, I and II, cloned upstream of the TATA box in the presence and absence of a downstream Tat-binding TAR element in a luciferase reporter vector) in the presence or absence of Tat plasmid and 100 ng of GRHAL1 RNA. The cells were harvested for luciferase assay 65 h after transfection. (**g**) Jurkat cells were co-transfected with 500 ng pGL3-SP1-Luc plasmid or pGL3-NFAT-Luc plasmid (**h**) in the presence or absence of 100 ng of GRHAL1 or GRHAL1-antisense control RNA. The cells were harvested for luciferase assay 65 h after transfection.

Because GRHAL1 activation of LTR-promoter-mediated transcription is enhanced by Tat, we next explored the roles of the Tat-binding TAR region (downstream of the LTR transcription start site), three Sp1 sites and two NF-κB sites (in the LTR core promoter) in GRHAL1-mediated LTR activation (Fig. 6a). To study the importance of these LTR regions in GRHAL1 function, we mutated pBlue3′LTR-luc plasmid and created a Tat-binding bulge deletion mutant plasmid, Del-B-LTR-Luc, in which TTT (+22 to +24) were deleted to abolish Tat binding. In addition, we generated two plasmids, Del-Sp1-LTR-Luc and Del-NF-κB-LTR-Luc, in which three Sp1-binding sites and two NF-κB sites in the core promoter region were deleted, respectively. Jurkat cells were transfected with pBlue3′LTR-luc-A or mutant plasmids and GRHAL1 RNA in the presence of a suboptimal concentration of Tat. As expected, the Jurkat cells transfected with pBlue3′LTR-luc-A plasmid, Tat and GRHAL1 RNA showed a robust induction of luciferase activity (Fig. 6b). Deletion of the Tat-binding bulge and Sp1-binding sites almost completely abolished the basal luciferase activity and the luciferase activity induced by a combination of Tat and GRHAL1 (Fig. 6c and d). In contrast, while deletion of NF-κB sites also strongly decreased the basal and Tat-induced luciferase activity, a combination of Tat and GRHAL1 RNA showed a significant induction of luciferase activity (Fig. 6e). Together, these data indicate that GRHAL1-mediated activation of the HIV-1 LTR is dependent on the promoter having an intact Tat-binding TAR region and Sp1-binding domains. This suggests that both Tat and Sp1 are essential for LTR-mediated transcription and act synergistically with GRHAL1 in mediating LTR activation, especially in conditions where the Tat concentrations are limiting.

To study the role of Sp1 and Tat synergy in a minimal LTR promoter setting, we cloned two LTR-specific Sp1 sites, I and II, upstream of TATA box in the presence and absence of a downstream Tat-binding TAR element in a luciferase reporter vector. Transfection studies with Jurkat cells indicated that the plasmid containing only Sp1 sites I and II without the TAR element had minimal basal activity and did not respond to Tat, GRHAL1 RNA or a combination of the two. This could be due to the exclusion of Sp1 site III that is known to compromise LTR function [26]. However, the plasmid that contained the two Sp1 sites and the TAR element showed basal activity and responded to both GRHAL1 RNA and Tat, and a combination of Tat and GRHAL1 RNA significantly enhanced the luciferase activity (Fig. 6f). These data further emphasize the importance of Tat and GRHAL1 synergy in mediating Sp1-dependent LTR activation and show that this synergy can also be reproduced in a minimal-promoter setting.

Our data have suggested that GRHAL1 also activates Tat-independent activity in HIV-1 LTR, implying that GRHAL1 can also function in the absence of Tat (Fig. 5c). To test whether GRHAL1 alone is sufficient to induce Sp1 activity, we used a plasmid, pGL3-SP1-Luc, that contained two generic Sp1-binding sites upstream of a TATA box. Jurkat cells transfected with pGL3-SP1-Luc in the presence of GRHAL1 RNA exhibited significant luciferase activity, whereas the GRHAL1-antisense RNA had no effect (Fig. 6g). In addition, GRHAL1 RNA had no effect on pGL3-NFAT-Luc used as control (Fig. 6g). Together, these data strongly indicate that GRHAL1 activates transcription by modulating Sp1 function and that GRHAL1 can activate Sp1-mediated transcription in non-HIV-LTR heterologous promoters, as well.

### Sp1 inhibitor mithramycin abrogates, whereas overexpression of Sp1 protein induces GRHAL1-mediated LTR activation

**Fig. 7.**
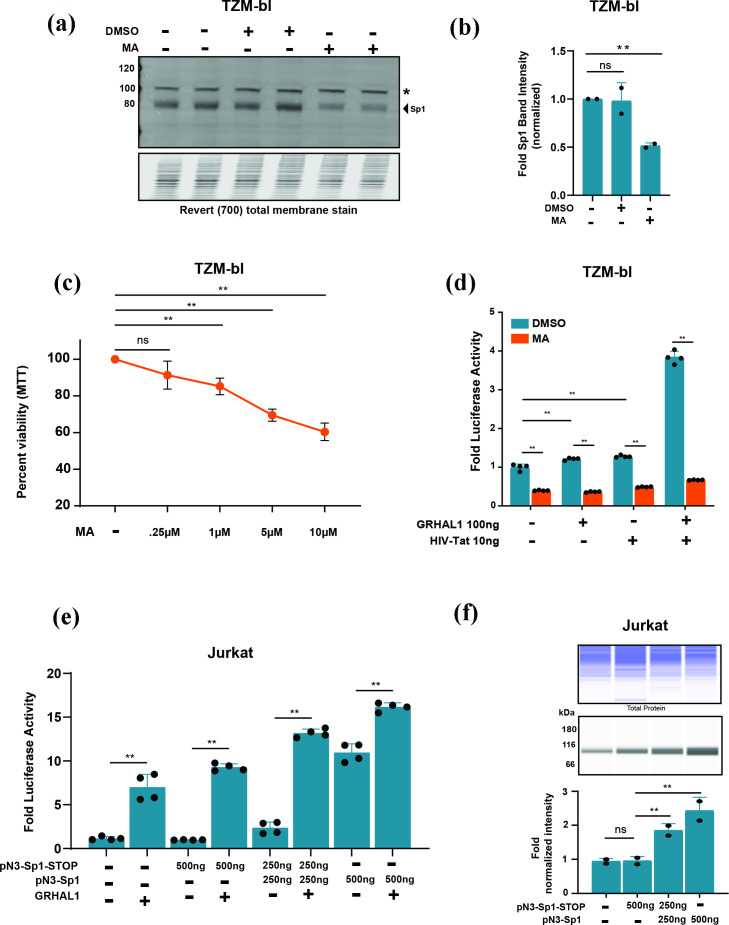
Effect of Sp1 inhibitor MA and overexpression of Sp1 protein on GRHAL1-mediated LTR activation. (**a**) TZM-bl cells (0.6 million) in six-well plates were treated in duplicate with 250 nM of MA or MA-matched volume of DMSO for 16 h and (**b**) subjected to Western blot using antibodies to Sp1 and the protein levels were quantitated after normalizing to total protein. (**c**) Cells were treated with indicated concentrations of MA for 16 h, and cell viability was quantitated by MTT assay. (**d**) TZM-bl cells (0.1 million) were transfected with 10 ng of Tat or 100 ng of GRHAL1 RNA or a combination of Tat and GRHAL1 RNA for 24 h, then treated with DMSO or 250 nM MA for 16 h before luciferase measurement. (**e**) Jurkat cells (0.25 million) were co-transfected with 0.25 µg pNL4-3.Luc.R-E plasmid in the presence of indicated concentrations of a control Sp1-non-expressing plasmid (pN3-Sp1-STOP) or Sp1 expressing plasmid (pN3-Sp1) and 100 ng of GRHAL1 for 65 h before luciferase measurement. (**f**) Western with anti-Sp1 antibody using Jess automated capillary-based Simple Western system using protein extracts from transfected cells showing a significant induction of Sp1 expression in pN3-Sp1-transfected cells as compared to cells transfected with pN3-Sp1-STOP plasmid.

To confirm the role of the Sp1 transcription factor in GRHAL1-mediated induction of HIV LTR, we tested the effect of MA, a selective inhibitor of the promoter-binding ability of Sp1 and known inhibitor of Sp1 expression [27,28], on basal and GRHAL1-mediated LTR activity. Treatment of TZM-bl cells with 250 nM MA showed that the inhibitor was able to reduce the levels of Sp1 (Fig. 7a and b), confirming previous findings that treating cells with MA inhibits Sp1 protein expression. MTT assay demonstrated that there was no significant effect of MA on the cell viability at 250 nM concentration (Fig. 7c). To study the effect of MA on LTR activity, we transfected TZM-bl cells with GRHAL1 RNA, with or without Tat, for 24 h, then treated them overnight with 250 nM MA. Fig. 7d shows that MA inhibited both the basal LTR activity and the LTR activity induced by the combination of Tat and GRHAL1 RNA.

To study the effect of overexpression of Sp1 protein on the basal and GRHAL1-mediated HIV-1 gene expression, Jurkat cells were co-transfected with pNL4-3.Luc.R-E plasmid in the presence of Sp1 expressing plasmid (pN3-Sp1) or a control Sp1-non-expressing plasmid (pN3-Sp1-STOP) and GRHAL1. The data showed a significant dose-dependent induction of luciferase activity in cells transfected with pN3-Sp1 plasmid as compared to control Sp1-non-expressing plasmid (Fig. 7e and f). While GRHAL1 transfection alone induced a significant increase in basal luciferase activity, cells transfected with GRHAL1 together with pN3-Sp1 plasmids showed enhancement of luciferase activity indicating synergy between GRHAL1 and Sp1 in mediating HIV-1 gene expression. Together, these data indicate that Sp1 activity is essential for LTR-mediated transcription and GRHAL1 function, and depletion or overexpression of Sp1 abrogates or potentiates both basal and GRHAL1-mediated HIV-1 gene expression, respectively.

### GRHAL1 interacts with Sp1, and Tat increases that interaction

**Fig. 8.**
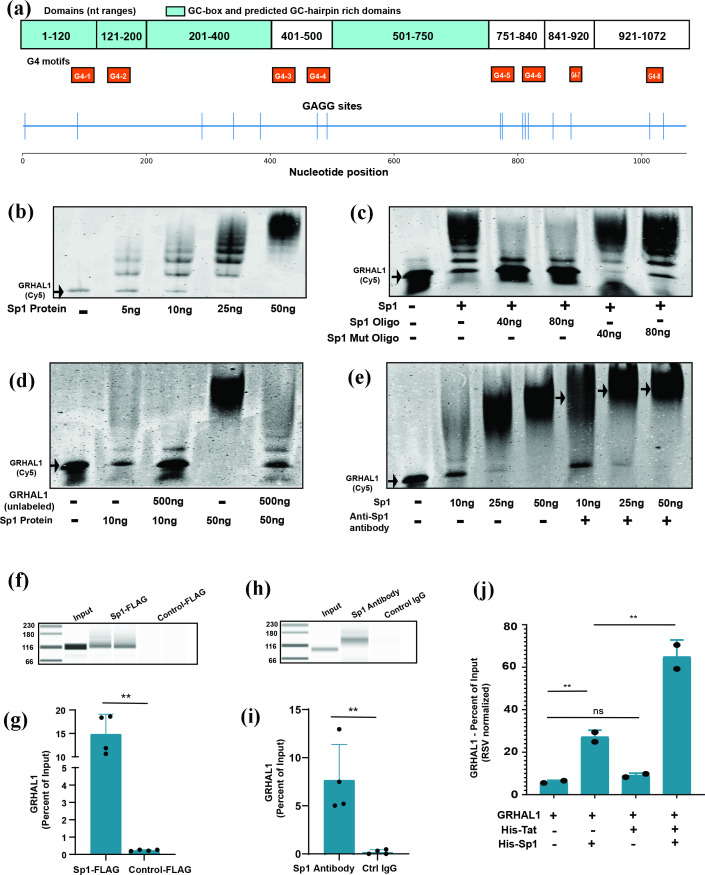
GRHAL1 interacts with Sp1 and Tat increases this interaction. (**a**) Schematic representation of putative Sp1-binding sites within GRHAL1 including GC boxes, GC-rich hairpin regions, G-quadruplex (g4) motifs and GAGG elements. (**b**) EMSA with 25 ng of Cy5-labelled GRHAL1 probe incubated with indicated concentrations of recombinant Sp1 protein. (**c**) EMSA with 25 ng of Cy5-labelled GRHAL1 probe and 50 ng of recombinant Sp1 protein and indicated competitors. (**d**) EMSA with 25 ng of Cy5-labelled GRHAL1 probe incubated with indicated concentrations of recombinant Sp1 protein. For the competition assay, 500 ng (25-fold excess) unlabeled GRHAL1 was used. (**e**) EMSA was performed with 25 ng of Cy5-labelled GRHAL1 probe incubated with indicated concentrations of recombinant Sp1 protein in binding buffer at 4 °C for 30 min. For super-shift assays, 2 µg of anti-Sp1 antibody (Millipore) was added for an additional 15 min at 4 °C. For all the EMSA assays, protein-bound probe was resolved on 1% agarose gels, then scanned at the 700 nm channel using a LI-COR Odyssey fluorescent scanner. (**f and g**) RIP assay, in which FLAG-tagged Sp1 or a FLAG-tagged non-Sp1 control plasmid was co-expressed with pcDNA-GRHAL1 plasmid in 293 FT cells, followed by isolation of the FLAG–Sp1–GRHAL1 complex from nuclear extracts using anti-FLAG antibody-conjugated magnetic beads. The bound Sp1 protein was pulled out, and Western analysis was performed using antibodies against FLAG peptide with the Jess automated capillary-based Simple Western system (**f**). GRHAL1 RNA fraction pulled out was quantitated by RT-PCR using GRHAL1-specific primers and Taqman probe. (**h and i**) RIP assay in which 293 FT cells were transfected with pcDNA-GRHAL1 plasmid followed by isolation of the endogenous Sp1 complexed with GRHAL1 from nuclear extracts using N-terminus-specific anti-Sp1 antibody conjugated to A/G magnetic beads. The bound Sp1 protein was pulled out, and Western analysis was performed with antibody to Sp1 (Millipore) using the Jess automated capillary-based Simple Western system (**h**). GRHAL1 RNA fraction pulled out was quantitated by RT-PCR using GRHAL1-specific primers and Taqman probe (**i**). **(j**) RNA-pulldown assay was carried out by spiking 2.5 µg of JNE with 500 ng of His-tagged recombinant Sp1 and 100 ng of GRHAL1 RNA in the presence or absence of 1 µg of His-tagged recombinant Tat. Complexes of GRHAL1 RNA with His-tagged protein were pulled down using His-Tagged Dynabeads™, and GRHAL1 RNA was quantitated by RT-PCR. For normalization, the eluants were spiked with equal amounts of RSV particles, then the RNA was isolated and subject to RT-PCR using GRHAL1- and RSV-specific primers.

lncRNAs are known to interact with cellular proteins and transcription factors and modulate gene transcription [29]. Recent findings have shown that Sp1 can bind RNAs in addition to its well-known DNA-binding activity [30]. We found many potential Sp1-binding sites that included GC boxes, GC hairpin-rich regions, G-quadruplexes (G4 motifs) and GAGG sites [29] (Fig. 8a) in GRHAL1 RNA and used EMSA, RIP and RNA-pulldown to study the binding of Sp1 protein to GRHAL1 RNA. Cy5-labelled GRHAL1 RNA interacted with purified recombinant Sp1 in EMSA (Fig. 8b). At a lower concentration of Sp1, the binding resulted in a ladder-like pattern or appeared as a smear, suggesting, as expected, that GRHAL1 has several Sp1 interaction sites. The specificity of the binding was confirmed by competition with a consensus ds Sp1-specific oligo (whereas a mutant ds Sp1 oligo did not compete) (Fig. 8c) and excess unlabeled GRHAL1 (Fig. 8d). Super-shift assay with an anti-Sp1 antibody confirmed that GRHAL1–Sp1 interaction was specific and was not due to any other protein that may be in the Sp1 protein preparation (Fig. 8e).

The interaction between Sp1 and GRHAL1 was further validated by RIP assays, in which FLAG-tagged Sp1 or a FLAG-tagged non-Sp1 control plasmid was co-expressed with pcDNA-GRHAL1 plasmid in 293 FT cells, followed by isolation of the FLAG–Sp1–GRHAL1 complex from nuclear extracts using anti-FLAG antibody-conjugated magnetic beads. The data show a nearly 15% enrichment of GRHAL1 bound to FLAG-Sp1 protein (Fig. 8f and g) compared to GRHAL1 input. Similar data were obtained in RIP assays with nuclear extracts from cells transfected with pcDNA-GRHAL1 plasmid alone and GRHAL1 complexed with endogenous Sp1 pulled out using antibody specific for amino acids 121–345 near the N-terminus of Sp1 protein (Fig. 8h and i). RIP eluates from endogenous Sp1 immunoprecipitation displayed decreased electrophoretic mobility of Sp1 by Western analysis (Fig. 8h). This is not unexpected, as RIP isolates native ribonucleoprotein complexes rather than monomeric proteins [31,32]. Consequently, the immunoprecipitated material may contain higher-order assemblies and post-translationally modified species that migrate at an increased apparent molecular weight. In addition to RIP, the interaction was also confirmed by RNA-pulldown assay in which JNE was spiked with His-tagged recombinant Sp1 in the presence of GRHAL1 RNA. The complex of GRHAL1 and His-tagged recombinant Sp1 was pulled down using Dynabeads His-Tag Isolation and Pulldown Kit, and GRHAL1 was quantitated by RT-PCR. There was a significant interaction of His-tagged Sp1 with GRHAL1 RNA (Fig. 8j). To study the effect of Tat on GRHAL1 and Sp1 interaction, we spiked JNE with GRHAL1 RNA and His-tagged recombinant Sp1 in the presence of His-tagged recombinant Tat, then pulled down GRHAL1 and His-tagged proteins and quantitated GRHAL1. His-tagged Tat alone did not pull down any GRHAL1, and thus Tat and GRHAL1 did not interact with each other. However, in the presence of His-tagged Sp1, Tat significantly increased the amount of GRHAL1 pulled down, indicating that the interaction between Sp1 and GRHAL1 was enhanced in the presence of Tat (Fig. 8j). Together, these data suggest that Sp1 interacts with multiple sites in GRHAL1 and Tat enhances the GRHAL1–Sp1 interaction.

## Discussion

We recently identified GADD34 as a restriction factor that inhibits HIV-1 replication [11]. GADD34, an ISG that is also induced during ISR signalling, functions primarily as a regulatory subunit of PP1 and forms a highly specific phosphatase holoenzyme capable of dephosphorylating eIF-2α, which restores protein homeostasis following endoplamic reticulum stress [33,36]. The role of GADD34 in regulating lncRNA function is unknown. In a gene array screen of GADD34-KO HeLa-CD4^+^ cells, we found ~2.5% of the lncRNAs were regulated by GADD34; of these, nearly 48% were upregulated and 52% were downregulated in KO cells. These data suggest an important role for GADD34 in lncRNAome regulation and point out the diversity of functional roles that GADD34 may perform by modulating lncRNA biology. We report the characterization of GRHAL1, a hitherto-uncharacterized lncRNA, and show that its expression is strongly upregulated in GADD34-KO cells. We found that GRHAL1 is an intergenic lncRNA located between the PCBP3 and COL6A1 genes. GRHAL1 is expressed in testis, along with a highly expressed isoform, LOC101928796. GRHAL1 is expressed, albeit at low levels, in several HIV-1-permissive cell lines, unlike the LOC101928796 isoform, which was not detected in these cell lines (Table 2S).

The functional role of GRHAL1 in CD4^+^ T cells is not known. Although its expression is low under normal physiological conditions, there is currently no evidence that GRHAL1 is important in maintaining the homeostasis in these cells. However, GRHAL1 expression is induced by TCR engagement and other T cell activation signals, indicating GRHAL1 may have a specialized physiological role during this cellular process. We show that GRHAL1 expression is significantly induced relatively early after HIV-1 infection and that increased levels are sustained for a week post-infection. The kinetics of GRHAL1 expression after HIV-1 infection point to a stimulatory role for this LncRNA in modulating HIV-1 replication. Interestingly, T cell activation signals that are known to favour HIV-1 replication [17,19] also induced GRHAL1 expression, validating a stimulatory role for GRHAL1 in HIV-1 biology. The finding that IFN treatment did not induce GRHAL1 expression suggests that GRHAL1 expression is an IFN-independent response and not a generalized IFN-mediated host innate immune response. Because treatment with the ISR inducer TN failed to induce GRHAL1 expression, ER-stress-mediated pathways also do not regulate GRHAL1 expression.

We have provided evidence of a stimulatory role of GRHAL1 in HIV-1 gene expression at the HIV-1 promoter level and shown that GRHAL1 induces both Tat-independent and Tat-dependent activation of both the unintegrated and integrated HIV-1 LTR promoter. Interestingly, the GRHAL1–Tat synergy to induce LTR activation was pronounced only at suboptimal levels of Tat, and the effect of GRHAL1 was lost at higher Tat concentrations. This could reflect the saturation of cellular response to two activating agents Tat and GRHAL1. This finding has important implications and illuminates how GRHAL1 may function, especially in the early stages of infection, where Tat concentrations in the cells are limited [37]. The role of GRHAL1 expression during the late stages of infection remains unclear. We speculate that its sustained expression may contribute to enhanced Tat-mediated transcriptional elongation at later time points, in addition to its apparent role in facilitating transcriptional initiation during the early phase of infection. Using mutational studies, we have shown that GRHAL1-mediated activation of HIV LTR was dependent on an intact Tat-binding TAR region and Sp1-binding promoter elements in the core promoter region. We have shown that the deletion of the Tat-binding bulge and Sp1-binding sites reduced the basal luciferase activity as well as the activity induced by a combination of Tat and GRHAL1. In contrast, while the deletion of NF-κB sites also reduced the basal and Tat-induced luciferase activity, a combination of Tat and GRHAL1 showed a significant induction of luciferase activity. These results suggest that both the Tat-binding region and Sp1 sites are critical for GRHAL1 function and that they act synergistically in mediating LTR activation, especially in conditions where the Tat concentrations are limiting. Interestingly, GRHAL1 was also found to induce the Tat-independent activity of HIV LTR, suggesting that GRHAL1 can also function in the absence of Tat, perhaps by inducing Sp1-dependent transcriptional activation. We found that GRHAL1 alone was sufficient to induce a non-LTR minimal Sp1 promoter but that it had no effect on a minimal NFAT promoter, indicating that GRHAL1 activates Tat-independent HIV LTR transcription by modulating Sp1 function and that GRHAL1 can activate Sp1-mediated transcription in heterologous promoters as well. Additional evidence that Sp1 is necessary for GRHAL1-mediated Tat-independent and Tat-dependent LTR activation came using MA, an inhibitor that blocks the binding of Sp1 to DNA promoter elements and reduces Sp1 levels. MA significantly inhibited the Sp1 levels and both the basal activity and the activity induced by a combination of Tat and GRHAL1. Together, these results confirm that GRHAL1-induced LTR activation is Sp1-dependent.

**Fig. 9.**
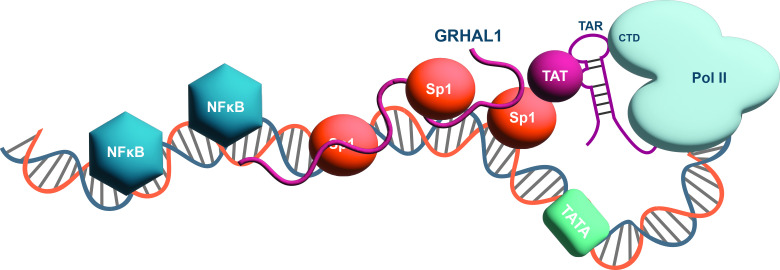
Hypothetical model for the activation of HIV-1 promoter by GRHAL1. GRHAL1 may associate with the HIV-1 LTR, potentially through RNA–DNA triplex formation within the promoter region or via RNA loop structures, thereby facilitating the recruitment of Sp1 in proximity to Tat. This spatial organization could promote a functional interaction between Sp1 and Tat, enhancing LTR activity. Such a mechanism may support HIV-1 replication under the conditions of limited Tat availability, particularly during the early stages of infection.

ncRNAs are known to interact with cellular proteins and transcription factors and modulate gene transcription by functioning as decoy, guide or scaffold or by triggering chromatin folding [38,49]. Recent findings have shown that Sp1 can bind RNAs in addition to its well-known DNA-binding activity [30]. We have identified multiple putative Sp1-binding sites within GRHAL1, including canonical GC boxes, GC-rich hairpin structures, G-quadruplex (G4) motifs and GAGG elements, suggesting a complex regulatory architecture for Sp1–lncRNA interaction. However, the specific contribution of each of these motifs to Sp1-binding affinity, structural stability and the functional activity of GRHAL1 remains to be elucidated. Future studies should focus on systematically dissecting these individual elements using mutational, structural and functional approaches to define their roles in mediating Sp1 recruitment and regulating GRHAL1-dependent transcriptional outcomes. Our data indicate that Sp1 interacts with GRHAL1 and modulates Tat- and GRHAL1-mediated HIV-1 LTR activation. This suggests a model in which, at suboptimal Tat levels, GRHAL1 may function as a guide to recruit Sp1 and Tat to the HIV LTR. Abolition of either Tat-binding or Sp1-binding sites in the LTR inhibits GRHAL1 function. We have not observed a direct interaction of GRHAL1 and Tat, but recombinant Tat significantly enhanced GRHAL1 and Sp1 interaction in an RNA-pulldown assay. We speculate that GRHAL1, perhaps by triplex formation with the HIV-1 LTR DNA in the promoter region or by formation of RNA loops, binds to the LTR and allows the recruitment of Sp1 in the vicinity of Tat, facilitating a functional interaction between Sp1 and Tat to modulate LTR function (Fig. 9). This promotes HIV-1 replication under the conditions of limited Tat levels, especially in the early stages of infection. Because GRHAL1 can also induce Sp1-mediated activation from a minimal promoter, it may be involved in yet-unknown Sp1-dependent cellular promoter activation functions. It is apparent that HIV-1 induces and hijacks cellular GRHAL1 to modulate Tat- and Sp1-dependent HIV-1 LTR activation and thus functions as an accessory factor to modulate HIV-1 replication. Our previous study [11] showed that HIV-induced GADD34 restricts viral replication at the level of translation, whereas the current study demonstrates that HIV-induced GRHAL1 enhances viral transcription. These findings indicate that GRHAL1 and GADD34 act at distinct stages – transcriptional and translational – forming a two-tiered regulatory mechanism during HIV infection.

## Supplementary material

10.1099/jgv.0.002288 Supplementary Material 1.

## Abbreviations

ds: double-stranded
EMSA: electrophoretic mobility shift assay
GADD34-KO: GADD34-knockout
GRHAL1: GADD34-regulated HIV accessory LncRNA-1
ION: ionomycin
ISG: interferon stimulated gene
ISR: integrated stress response
JNE: Jurkat nuclear extract
KI: knock-in
KO: knockout
lncRNA: long non-coding RNA
MA: mithramycin A
MTT: 3-(4,5-dimethylthiazol-2-yl)−2,5-diphenyltetrazolium bromide
PMA: phorbol 12-myristate 13-acetate
RACE: rapid amplification of cDNA end
RIP: RNA immunoprecipitation
RSV: Rous sarcoma virus
RT-PCR: real-time polymerase chain reaction
TCR: T cell receptor
TN: tunicamycin
VSV: vesicular stomatitis virus
