# Supplementary material for: GRHAL1, a novel lncRNA, regulates HIV-1 gene expression by modulating Tat- and Sp1-mediated HIV-1 LTR activation
Source: J Gen Virol. 2026 Jul 2;107(7):002288. doi: 10.1099/jgv.0.002288 (PMC13334546; doi:10.1099/jgv.0.002288)
Supplement: Supplementary Material 1. [file jgv-107-02288-s001.pdf]

1-S. https://doi.org/10.6084/m9.figshare.32204967

Fig. 1-S. Gene Array Data (ArrayStar) showing upregulated and downregulated lncRNAs in GADD34-KO HeLa-CD4+ cells compared to wild-type HeLa-CD4+ cells.

2-S.

	RT-PCR Result	
	GRHAL1	LOC101928796
**Testis**	Present	Present
**Jurkat**	Present	Undetected
**MT-2**	Present	Undetected

Table 2-S. RT-PCR for GRHAL1 and LOC101928796 in Testis, Jurkat, and MT-2 cell lines. 2 ug RNA was reverse-transcribed using SuperScript IV reverse transcriptase (Thermo Fisher Scientific) with 2.5 μM random hexamers. cDNA was analyzed by RT-PCR using gene-specific primers (Table 1) and normalized to RNase P as described in the Methods.

3-S.

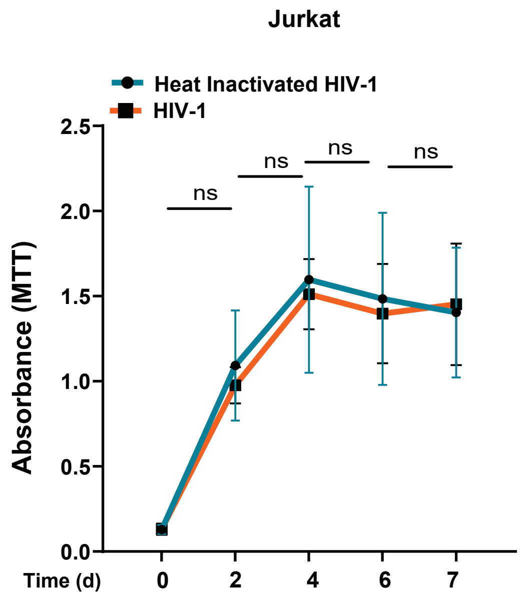

Fig. 3-S. Jurkat cells infected with HIV-1 or heat-inactivated HIV-1 were seeded in 96-well plates at the indicated time points, and cell viability was assessed using an MTT-based assay as described in the Methods. The data is presented as absorbance values measured at 570nm, compared to cells infected with heat-inactivated HIV-1.

5-S1.

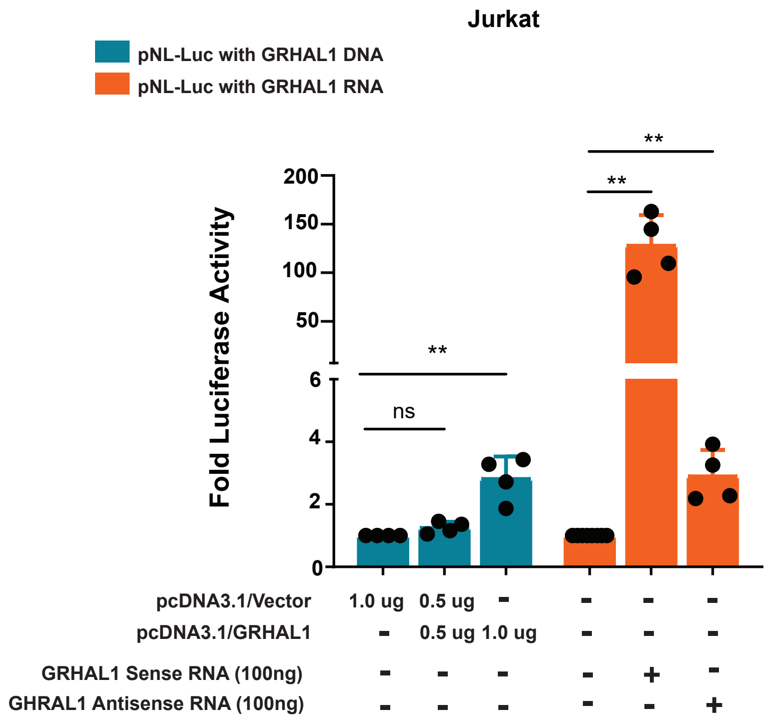

5-S2.

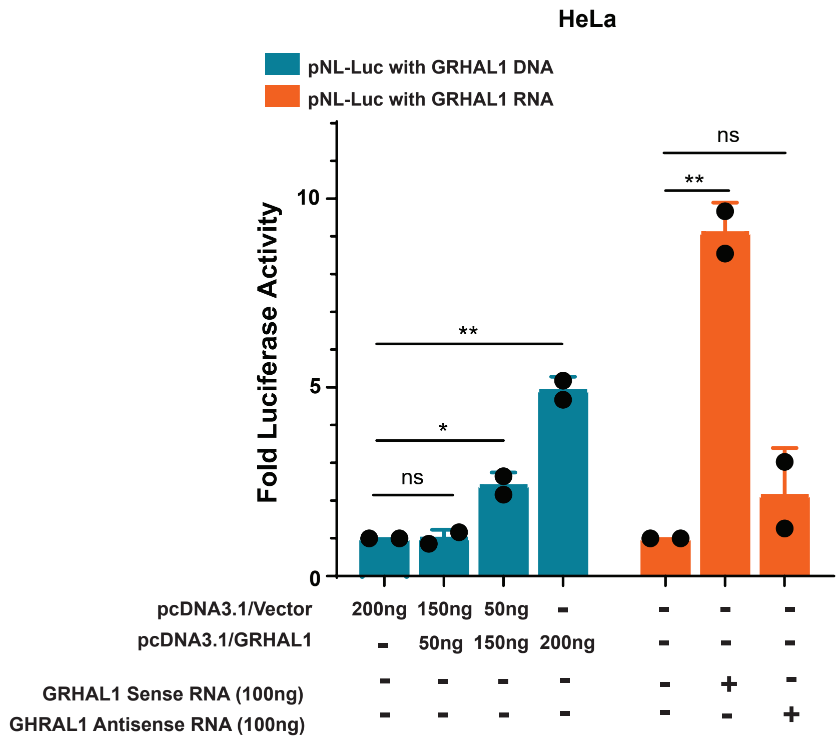

5-S3.

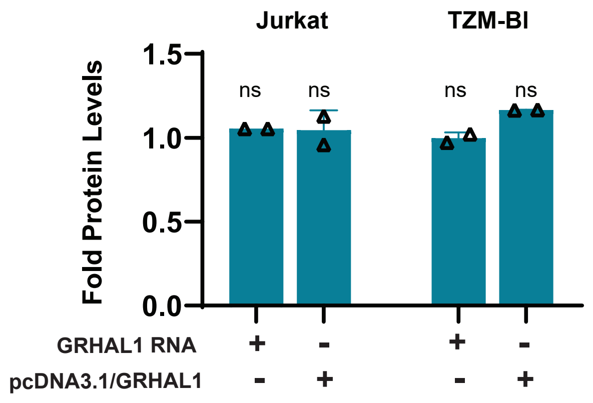

Fig. 5-S1-S2 and 5-S3. Cotransfection of Jurkat cells with 0.25ug pNL4-3.Luc.R-E- plasmid (Fig. 5-S1) and HeLa cells with 0.1ug of pNL4.3.Luc.R-E plasmid (Fig. 5-S2) in the presence of indicated concentrations of in-vitro-synthesized GRHAL1 or GRHAL1 antisense RNA or pcDNA3.1/GRHAL1 plasmid. Cells were harvested for luciferase activity quantitation 65h after transfection in Jurkat cells and 36h after transfection in HeLa cells. (Fig. 5-S3) Comparison of protein levels in cellular lysates from Jurkat and TZM-BI cells under the conditions of the RNA and DNA transfections. Shown here are the protein levels from Jurkat cells transfected with 0.25ug pNL4.3.Luc.R-E- plasmid in the presence of 100ng of GRHAL1 or 1.0ug of pcDNA3.1/GRHAL1 plasmid for 65h and protein levels from TZM-BI cells transfected with 100ng of GRHAL1 or 1.0ug of pcDNA/GRHAL1 plasmid for 36h.
Total protein concentrations were quantitated using BCA assay.
